# Selenoprotein F Knockout Caused Glucose Metabolism Disorder in Young Mice by Disrupting Redox Homeostasis

**DOI:** 10.3390/antiox11112105

**Published:** 2022-10-25

**Authors:** Min Li, Yun Zhang, Jun Zhou, Hongmei Liu

**Affiliations:** 1 Hubei Key Laboratory of Bioinorganic Chemistry and Materia Medica, School of Chemistry and Chemical Engineering, Huazhong University of Science and Technology, Wuhan 430074, China; 2 College of Chemistry and Molecular Sciences, Wuhan University, Wuhan 430072, China; 3 Shenzhen Huazhong University of Science and Technology Research Institute, Shenzhen 518057, China

**Keywords:** selenoprotein F, knockout, glucose metabolism, oxidative stress

## Abstract

Selenoprotein F (SELENOF) might play an important role in maintaining human health since an increasing number of studies have linked SELENOF deficiency to various pathologies such as cancer and neurodegeneration. We have previously reported on glucose metabolism disorders in SELENOF knockout mice, which imply a novel biological function of SELENOF in glucose metabolism. However, the underlying mechanism and whether the effect of SELENOF on glucose metabolism is age-dependent remain unknown. In the present study, we compare the metabolic phenotype in more detail as well as the oxidative stress parameters in SELENOF knockout mice (C57BL/6J background) and naïve C57BL/6J mice of different ages (12, 16 and 21 weeks old). The results showed that SELENOF knockout caused glucose metabolism disorders only in young mice, especially in 12-week-old mice, characterized by hyperglycemia, serum insulin reduction, impaired glucose tolerance, decreased insulin sensitivity, decreased glucose catabolism, increased gluconeogenesis and impaired insulin signaling pathway. These abnormalities gradually improved with age and disappeared in knockout mice at 21 weeks old. Furthermore, before 16 weeks old, SELENOF knockout mice showed increased lipid peroxidation and decreased glutathione/glutathione disulfide ratio and glutathione peroxidase activity in the serum and liver. Furthermore, the expression of glutathione peroxidase 1 significantly reduced in the liver and pancreas. Our findings suggest that SELENOF knockout might cause glucose metabolism disorders in young mice via the disruption of redox homeostasis.

## 1. Introduction

As one of the main sources of energy supply for the body, glucose metabolism plays a key role in life activities. Normal glucose metabolism is very important for the functioning of the body [1,2]. However, with changes in lifestyle and dietary habits, glucose metabolism disorders frequently occur in people of different ages and often lead to diseases such as obesity and diabetes mellitus, which have great adverse impacts on human health and quality of life [3,4].

Selenium (Se), as an essential trace element, is implicated in many facets of human health and diseases, including cardiovascular disease, cancer and diabetes. The role of Se in diabetes is still a matter of discussion with respect to both Se deficiency and Se supranutrition [5,6,7]. Se may have a dual role, both as a protective factor and as a risk factor of diabetes, depending on its basal plasma concentration and the individual’s diet intake [6,8]. A number of epidemiological studies have shown that an inadequate intake of Se is associated with an increased incidence of diabetes [9,10,11]. For example, a study conducted on the elderly French population found the incidence of type 2 diabetes (T2DM) was greater in men with lower median plasma Se concentrations compared to those with higher concentrations [9]. Moreover, many experimental studies have reported that dietary Se deficiency induces T2DM-like symptoms (i.e., glucose intolerance and insulin resistance) in mice, which might provide explanations for the association of Se deficiency and the risk of T2DM observed in humans [12,13]. All these results indicate that Se is necessary for maintaining normal glucose metabolism.

The biological role of Se is principally mediated by selenocysteine (Sec) that is incorporated into 25 selenoproteins in humans [14]. However, dietary Se deficiency does not equally reduce the expression of all selenoproteins. The expression of low-hierarchy selenoproteins, including glutathione peroxidase 1 (GPX1), selenoprotein H (SELENOH), selenoprotein W (SELENOW) and selenoprotein F (SELENOF) are significantly downregulated in Se-deficient mice [13,15]. The decreased expression of these low-hierarchy selenoproteins may be one of the reasons why Se deficiency caused T2DM-like symptoms in mice [13]. For instance, GPX1 knockout in mice led to the disruption of glucose metabolism homeostasis, as demonstrated by hypotrophy of pancreatic islet β-cells, insulin hyposecretion and hypoinsulinemia [16]. However, as SELENOF is one of the low-hierarchy selenoproteins, whether it is implicated in glucose metabolism remains unclear.

SELENOF is an endoplasmic reticulum (ER)-resident protein with a C-terminal thioredoxin-like domain, a key structure rendering SELENOF a potential thiol-disulfide oxidoreductase in the ER [17]. SELENOF might participate in the quality control process of protein folding by binding the UDP-glucose: glycoprotein glucosyltransferase (UGGT), an ER-resident chaperone protein, but the specific mechanism has not been uncovered until now [17,18,19,20]. In addition, many studies have reported that SELENOF has a correlation with various diseases, including several types of cancers (e.g., breast cancer, colon cancer and prostate cancer), acquired immune deficiency syndrome (AIDS), and neurodegeneration diseases [17], suggesting that SELENOF is closely related to human health. For example, a significant association between SELENOF genetic variations and elevated breast cancer risk in African-American women has been observed [21]. However, little is known about the relationship between SELENOF and glucose metabolism.

In our previous works, SELENOF knockout (KO) mice (C57BL/6J background) were constructed using clustered regularly interspaced short palindromic repeats (CRISPR) and the CRISPR-associated protein 9 (Cas9) gene editing method. Compared with naïve C57BL/6J mice, SELENOF KO mice showed the differential expression of hepatic proteins involved in the glucose metabolic pathways, glucose intolerance and insulin reduction, suggesting that glucose metabolism disorders were induced by SELENOF KO [22,23]. Furthermore, we observed by accident that SELENOF KO mice had an impaired glucose tolerance at 2.5 months and 4 months old, but the difference disappeared when KO mice were 5 months old [22], suggesting that SELENOF might affect glucose metabolism in young mice, but more details have not yet been obtained.

In order to clarify whether the effect of SELENOF on glucose metabolism is age-dependent and to investigate the underlying mechanism, we analyzed the metabolic phenotype, the expression levels of key enzymes involved in glucose metabolic pathways, the insulin signaling pathway, and the oxidative stress parameters in both KO and naïve C57BL/6J mice of different ages (12, 16 and 21 weeks old). This will not only help us further understand the function of SELENOF and its role in the development of glucose metabolism-related diseases but will also help us find new potential therapeutic targets for such diseases.

## 2. Materials and Methods

### 2.1. Materials

Polyvinylidene fluoride (PVDF) membrane and the enhanced chemiluminescence (ECL) kit were purchased from Millipore (Billerica, MA, USA). Primary antibodies against Cu/Zn superoxide dismutase (SOD1), GPX1, protein kinase B (AKT), phospho- AKT (p-AKT), forkhead box O1 (FoxO1), phospho- FoxO1 (p-FoxO1), glycogen synthase kinase-3β (GSK3β), phospho- GSK3β (p-GSK3β), calnexin (CNX) and glucose regulated protein 78 kD (GRP78) were purchased from Wanleibio (Shenyang, China). Primary antibodies against UGGT, SELENOF, selenoprotein T (SELENOT) and selenoprotein S (SELENOS) were purchased from Abcam (Cambridge, UK). The primary antibody against thioredoxin reductase 1 (TXNRD1) was purchased from Boster (Wuhan, China). The Hematoxylin and Eosin (HE) Staining Kit, Radio-immunoprecipitation assay (RIPA) lysis buffer, phenylmethanesulfonylfluoride (PMSF), primary antibodies against glucose 6-phosphate dehydrogenase (G6PD), fructose-1,6-bisphosphatase (FBP), GAPDH, β-Tubulin and β-actin were purchased from Beyotime (Shanghai, China). The primary antibody against catalase (CAT) was purchased from GeneTex (Irvine, CA, USA). The primary antibody against phosphofructokinase (PFK) was obtained from Santa Cruz, TX, USA. The cat. numbers of all primary antibodies are listed in Table S1. Horseradish peroxidase (HRP)-conjugated secondary antibodies were purchased from Biosharp (Hefei, China). The assay kits for malondialdehyde (MDA), reduced glutathione (GSH) and its oxidized form (glutathione disulfide, GSSG) levels and the assay kits for the activities of total SOD (T-SOD), CAT and GPX were purchased from Elabscience (Wuhan, China). The Glycogen kit was purchased from NanJing JianCheng Bioengineering Institute (Nanjing, China). The Insulin ELISA kit was obtained from CUSABIO (Wuhan, China). All chemicals from Sinopharm chemical reagent Co., Ltd. were of analytical grade and used without further purification.

### 2.2. Animals

Thirty male naïve C57BL/6J mice (7 weeks old) were purchased from the China Three Gorges University Research Center for Laboratory Animals (Yichang, Hubei, China) and adaptive fed for 1 week. Then they were randomly divided into 3 groups (10 mice in each group) and fed up to 12, 16 and 21 weeks old. The SELENOF KO C57BL/6J mice generated using the CRISPR/Cas9 technique were bred in our laboratory and were identified by polymerase chain reaction (PCR), agarose gel electrophoresis and Western blot as described in our previous study [23]. Detailed information on the identification of SELENOF KO mice is provided in Figure S1. Thirty male KO mice of the same age as C57BL/6J mice were also grouped and fed for the same amount of time. All animals were housed in cages (5 mice per cage) in a room kept at 22–25 °C under a 12-h light/dark cycle and were provided with adequate water and a standard diet ad libitum.

The body weight of each mouse was recorded weekly. The fasting blood glucose level test, glucose tolerance test (GTT) and insulin tolerance test (ITT) were carried out 1 or 2 weeks before the mice were sacrificed. After the mice were sacrificed, the serum and tissue samples were collected and stored at −80 °C for further studies. The schematic design of animal experiments is shown in Scheme S1. All the animal procedures were approved by the Institutional Animal Care and Use Committee, Huazhong University of Science and Technology (protocol code: 2701; date of approval: 1 October 2018).

### 2.3. Fasting Blood Glucose Level Test, GTT and ITT

When the mice were 10, 15 and 20 weeks old, the fasting blood glucose level test and GTT were performed on Monday and ITT was performed on Saturday. For the fasting blood glucose level test and GTT, mice were fasted overnight for 12 h. Blood was then collected from the tail and subjected to glucose measurements at 0 min (baseline, also fasting blood glucose level) and then at 15, 30, 60, 90, and 120 min after an oral administration of glucose (1 g/kg body weight). For the ITT, mice fasted for 4 h in the daytime were injected intraperitoneally with insulin (0.8 IU/kg body weight). Blood was collected from the tail and subjected to glucose measurements at 0, 15, 30, 60, 90, and 120 min. Once the blood glucose level of the mouse was abnormal (the blood glucose value exceeded the detection range of the glucometer after the injection of glucose or hypoglycemia occurred after the injection of insulin), the data of the mouse would be discarded.

### 2.4. Measurements of Serum and Hepatic Biochemical Parameters

To determine the oxidative stress parameters of the liver, the liver tissue was weighed and homogenized with phosphate buffer saline (PBS) at a ratio of 1:9. The supernatant was obtained by centrifugation at 12,000 rpm for 15 min at 4 °C. The contents of MDA, GSH and GSSG as well as the activities of T-SOD, GPX, and CAT in liver homogenate or serum were analyzed using commercial kits. The serum insulin levels were detected using a commercial ELISA kit according to the manufacturer’s instructions.

### 2.5. Liver and Muscle Glycogen Determination

Glycogen content testing was carried out according to the manufacturer’s instructions using a commercial kit. Briefly, liver or muscle tissues were rinsed with saline, blotted dry on filter paper and weighed. Alkaline solution was added at a ratio of 1:3 according to the sample weight and the mixtures were hydrolyzed in boiling water for 20 min. After cooling with running water, the hydrolyzed solution was diluted to form a glycogen detection solution, which was then subjected to a color reaction with anthrone. Finally, quantitative calculation was performed according to the absorbance at 620 nm determined by a spectrometer (Tecan, infinite 200, Zurich, Switzerland).

### 2.6. Western Blot Analysis

Liver or pancreas tissues were homogenized with RIPA lysis buffer containing 1 mM PMSF and centrifuged at 12,000 rpm for 15 min at 4 °C. The supernatants were collected and the protein concentrations were measured using the Lowry method [24]. Protein samples with unified concentrations were separated by sodium dodecyl sulfate polyacrylamide gel electropheresis (SDS-PAGE), and then transferred to PVDF membranes. The membranes were blocked with TBS-T solution containing 5% bovine serum albumin or non-fat milk and then incubated overnight with primary antibodies. After extensive washing, the membranes were incubated with HRP-conjugated secondary antibody and visualized with ECL kit using a Tanon 5200 MultiImage System (Tanon, Shanghai, China). The densities of the protein bands were quantified by ImageJ software (v1.53i, National Institutes of Health, Bethesda, MD, USA).

### 2.7. Histopathology of the Liver and Pancreas

Histopathological analysis of the liver and pancreas was performed by hematoxylin-eosin (HE) staining.

### 2.8. Statistical Analysis

Results from a representative of at least three independent experiments are shown as mean ± SD. Statistical analysis was performed using SPSS 23.0 (IBM, Armonk, NY, USA). For a comparison of the two data sets from C57BL/6J mice and SELENOF KO mice of the same age, Student’s *t*-test was used. A value of *p* < 0.05 was considered statistically significant.

## 3. Results

### 3.1. Metabolic Phenotype Analysis of SELENOF KO Mice

**Figure 1:**
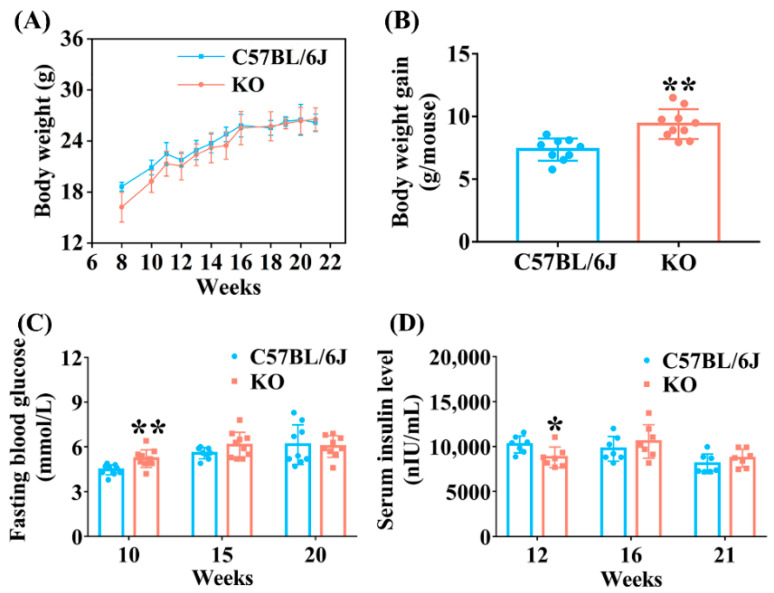
Metabolic phenotype analysis of selenoprotein F (SELENOF) knockout (KO) mice. (**A**) Body weight (*n* = 10). (**B**) Body weight gain of mice aged 8 to 16 weeks (*n* = 9 for C57BL/6J mice and *n* = 10 for KO mice). (**C**) Fasting blood glucose level (*n* = 10 at week 10, *n* = 8 for C57BL/6J mice and *n* = 10 for KO mice at week 15, *n* = 9 at week 20). (**D**) Serum insulin level (*n* = 7). Data were expressed as mean ± SD. * *p* < 0.05, ** *p* < 0.01, compared with C57BL/6J mice.

As functional carriers of Se, many studies have reported that selenoprotein deficiency or overexpression may affect growth status [25,26]. Therefore, we first detected the body weight changes of KO and C57BL/6J mice. When at 8 weeks old, the body weight of KO mice was significantly lower than that of C57BL/6J mice under the same conditions. After that, the body weight of KO mice increased rapidly and reached the same level of C57BL/6J mice at 16 weeks old. There was no difference in body weight between KO and C57BL/6J mice aged 16 to 21 weeks (Figure 1A). The net body weight gain of KO mice aged 8 to 16 weeks was significantly higher than that of C57BL/6J mice (Figure 1B), suggesting that SELENOF KO might lead to metabolic abnormalities in young mice.

To explore the effect of SELENOF KO on glucose metabolism, the fasting blood glucose and serum insulin levels of the mice were analyzed. Fasting blood glucose levels increased significantly in KO mice at 10 weeks old compared with age-matched C57BL/6J mice, but there was no difference between KO and C57BL/6J mice at 15 weeks and 20 weeks old (Figure 1C). Moreover, SELENOF KO mice displayed significantly decreased fasting serum insulin levels compared with C57BL/6J mice at 12 weeks old, and this difference disappeared when mice were 16 weeks and 21 weeks old (Figure 1D). Taken together, these results demonstrate that SELENOF KO led to hyperglycemia and serum insulin reduction in young mice and serum insulin reduction might have been one of the causes of hyperglycemia.

### 3.2. SELENOF KO Led to Impaired Glucose Tolerance and Decreased Insulin Sensitivity in Young Mice

**Figure 2:**
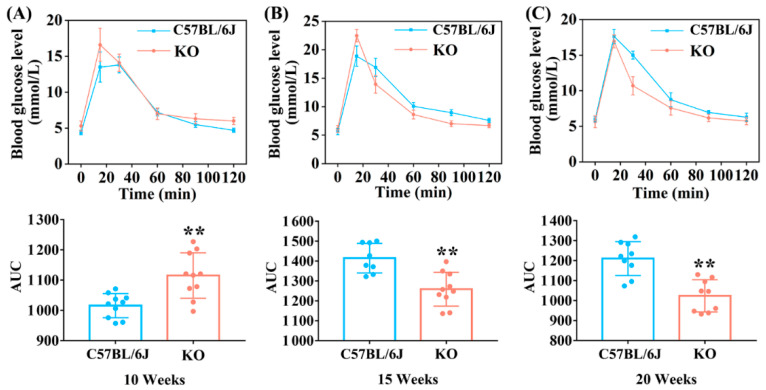
GTT (Glucose tolerance test) of SELENOF KO and C57BL/6J mice at different ages. (**A**) GTT detected at 10 weeks (*n* = 10). (**B**) GTT detected at 15 weeks (*n* = 8 for C57BL/6J mice and *n* = 10 for KO mice). (**C**) GTT detected at 20 weeks (*n* = 9). Area under curve (AUC) value was calculated. Data were expressed as mean ± SD. ** *p* < 0.01, compared with C57BL/6J mice.

GTT is a common method used to evaluate glucose tolerance status that can provide information about insulin resistance and insulin secretion both directly and indirectly [27]. According to the results of GTT shown in Figure 2A, it was clear that glucose tolerance was impaired in KO mice compared with C57BL/6J mice at 10 weeks old. At 15 weeks old, although the peak value of blood glucose level at 15 min in KO mice was significantly higher than that in C57BL/6J mice, the glucose level dropped sharply at the subsequent time points and the area under curve (AUC) value was also conspicuously lower than that of C57BL/6J mice, indicating an improvement in impaired glucose tolerance in KO mice (Figure 2B). Glucose tolerance was significantly improved in KO mice at 20 weeks old since the blood glucose levels remained lower at all time points except 15 min compared with C57BL/6J mice (Figure 2C). These data showed that SELENOF KO significantly impaired glucose tolerance in young mice, which might be attributed to the decreased serum insulin level, as shown in Figure 1D.

**Figure 3:**
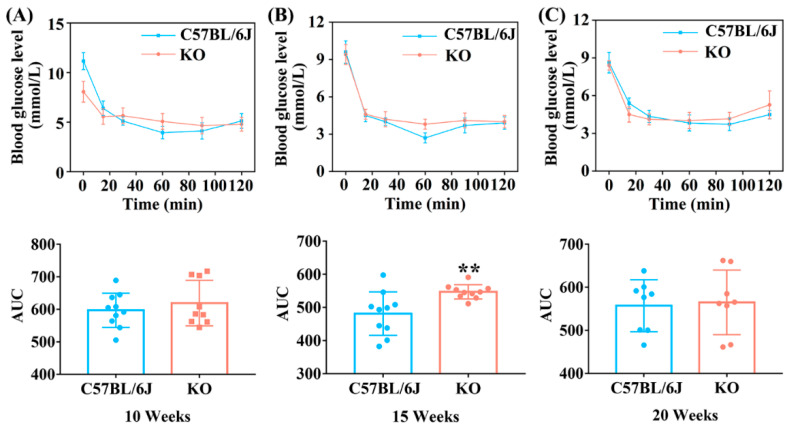
ITT (Insulin tolerance test) of KO and C57BL/6J mice at different ages. (**A**) ITT detected in 10 weeks (*n* = 10 for C57BL/6J mice and *n* = 9 for KO mice). (**B**) ITT detected in 15 weeks (*n* = 10). (**C**) ITT detected in 20 weeks (*n* = 8). Data were expressed as mean ± SD. ** *p* < 0.01, compared with C57BL/6J mice.

ITT is used to evaluate the activity of insulin and insulin sensitivity of the body [28]. Thus, we further analyzed the ITT of mice at different ages. At the age of 10 weeks, although the fasting blood glucose level of C57BL/6J mice was much higher than that of KO mice at 0 min (possibly caused by unconsumed food remaining in the gut), it decreased rapidly after insulin injection and dropped to a lower level than that in KO mice after 30 min (Figure 3A). At the age of 15 weeks, the blood glucose level of KO mice remained higher than that of C57BL/6J mice after 15 min of insulin injection (Figure 3B). These data showed that KO mice were less sensitive to insulin at 10 and 15 weeks old. However, the insulin sensitivity of KO mice was the same as that of C57BL/6J mice at 20 weeks old, since the change in trend of blood glucose level in KO mice showed no significant difference compared with C57BL/6J mice after insulin injection (Figure 3C). The above results suggest that SELENOF KO led to decreased insulin sensitivity in young mice.

### 3.3. Effect of SELENOF KO on the Expression of Key Enzymes Involved in Glucose Metabolism Pathways

**Figure 4:**
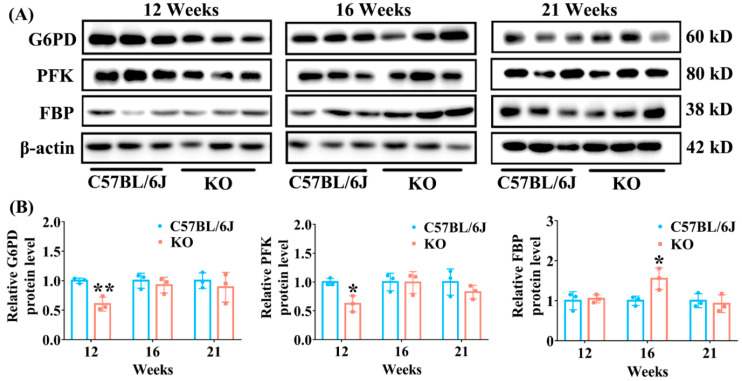
The expression of enzymes involved in glucose metabolism pathways in the liver of KO and C57BL/6J mice of different ages. (**A**) Representative Western blot bands of G6PD, PFK and FBP. (**B**) Semi-quantification analysis of G6PD, PFK and FBP normalized to β-actin. G6PD: glucose 6-phosphate dehydrogenase; PFK: phosphofructokinase; FBP: fructose-1,6-bisphosphatase. Data were expressed as mean ± SD (*n* = 3). * *p* < 0.05, ** *p* < 0.01, compared with C57BL/6J mice.

Decreased insulin leads to abnormal glucose metabolism pathways, which further results in hyperglycemia. Therefore, we next analyzed the expression of key enzymes involved in glucose metabolism pathways in the livers of SELENOF KO and C57BL/6J mice of different ages. G6PD is a key enzyme involved in the pentose phosphate metabolism pathway and PFK is a rate-limiting enzyme involved in the glycolytic pathway. As shown in Figure 4, compared with C57BL/6J mice, the levels of G6PD and PFK were significantly decreased in the livers of KO mice at 12 weeks old. But this difference disappeared when mice were 16 and 21 weeks old. The decreased expression of these two enzymes will cause the body’s blood glucose to rise. Furthermore, we found that the expression of FBP, a key enzyme in the gluconeogenesis pathway, was significantly increased in KO mice at 16 weeks of age, which further contributed to high blood glucose. Collectively, these results suggest that SELENOF KO caused the decreased glucose catabolism and the increased gluconeogenesis in mice younger than 16 weeks of age, which might have partially contributed to hyperglycemia.

### 3.4. SELENOF KO Reduced Glycogen Accumulation in the Liver and Muscle of Mice at an Earlier Age

**Figure 5:**
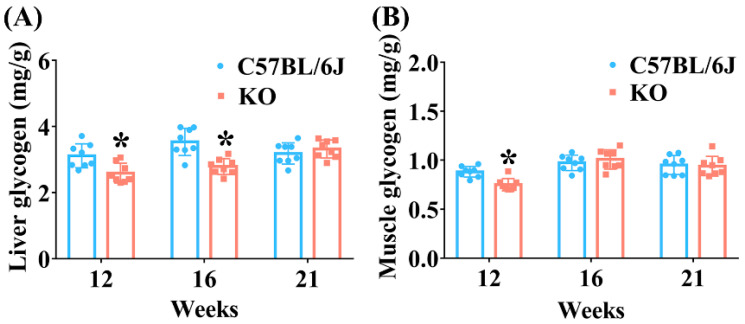
Glycogen contents in the liver (**A**) and muscle (**B**) of SELENOF KO and C57BL/6J mice at different ages. Data were expressed as mean ± SD (*n* = 8). * *p* < 0.05, compared with C57BL/6J mice.

Glycogen is a polysaccharide mainly stored in skeletal muscle and the liver in mammals, and it is an important indicator of glucose metabolism associated with glucose distribution and uptake [29]. Insulin can stimulate peripheral tissue (such as skeletal muscle and liver) to assimilate glucose and increase their ability to synthesize glycogen. Consistent with the decreased serum insulin level in SELENOF KO mice of 12 weeks old, the glycogen content significantly decreased in both the liver and muscle tissues of KO mice aged 12 weeks (Figure 5). The glycogen content also significantly decreased in the liver of KO mice at 16 weeks old. These data indicate that the decreased accumulation of glycogen in the liver and muscle might be another cause of hyperglycemia in young SELENOF KO mice.

### 3.5. SELENOF KO Resulted in Inhibition of AKT Signaling Pathway in Young Mice

**Figure 6:**
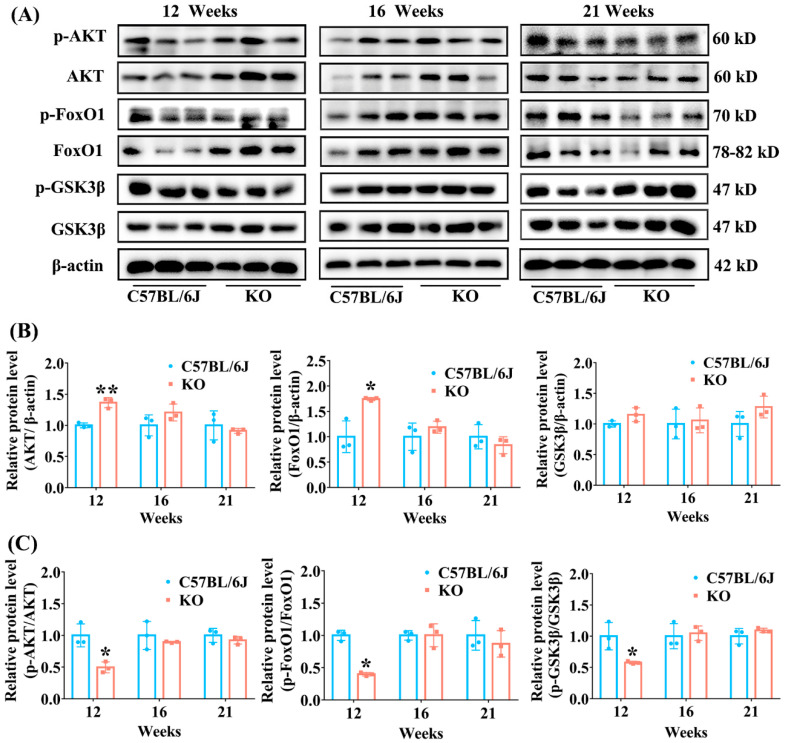
Effect of SELENOF KO on the expression of key proteins involved in insulin signaling pathway in the liver of mice at different ages. (**A**) Representative Western blot bands of phosphorylated AKT (p-AKT), AKT, phosphorylated FoxO1 (p-FoxO1), FoxO1, phosphorylated GSK3β (p-GSK3β) and GSK3β. (**B**) Semi-quantification analysis of AKT, FoxO1 and GSK3β normalized to β-actin. (**C**) Semi-quantification analysis of p-AKT normalized to AKT, p-FoxO1 normalized to FoxO1, and p-GSK3β normalized to GSK3β. AKT: protein kinase B; p-AKT: phospho- AKT; FoxO1: forkhead box O1; p-FoxO1: phospho- FoxO1; GSK3β: glycogen synthase kinase-3β; p-GSK3β: phospho- GSK3β. Data were expressed as mean ± SD (*n* = 3). * *p* < 0.05, ** *p* < 0.01, compared with C57BL/6J mice.

The above results of GTT and ITT demonstrated that SELENOF KO mice showed decreased insulin sensitivity, which is one of the manifestations of insulin resistance. The AKT-FoxO1/GSK3β signaling pathway is very important in enabling the body to use insulin to regulate glucose metabolism. Impaired AKT-FoxO1/GSK3β signal transduction is often accompanied by impaired glucose tolerance and insulin sensitivity [30,31]. Therefore, we further explored the level of AKT, FoxO1 and GSK3β involved in the insulin signaling pathway in the liver. The results showed that the relative levels of unphosphorylated AKT and FoxO1 in SELENOF KO mice increased by 36% (*p* < 0.01) and 74% (*p* < 0.05) when normalized to β-actin, respectively, while the level of unphosphorylated GSK3β had no change, compared with C57BL/6J mice at 12 weeks old (Figure 6B). The increase in unphosphorylated AKT and FoxO1 resulted in a significant decrease in p-AKT (reduced by 50% when normalized to unphosphorylated AKT) and p-FoxO1 (reduced by 60% when normalized to unphosphorylated FoxO1) in KO mice at 12 weeks old. Also, the p-GSK3β level when normalized to unphosphorylated GSK3β was significantly decreased in KO mice at 12 weeks old (Figure 6C). By contrast, SELENOF KO had no effect on the levels of unphosphorylated AKT, FoxO1 and GSK3β when normalized to β-actin or on the levels of phosphorylated AKT, FoxO1 and GSK3β when normalized to the corresponding unphosphorylated protein in mice at 16 and 21 weeks old, in comparison with C57BL/6J mice (Figure 6). These results indicate that SELENOF KO might lead to decreased insulin sensitivity to some extent by inhibiting the AKT signaling pathway in young mice. 

### 3.6. SELENOF Knockout Resulted in the Disruption of Redox Homeostasis in Young Mice

Serum oxidative stress parameters, including lipid peroxidation, GSH, GSSG, a GSH:GSSG ratio (GSH/GSSG), activities of GPX, T-SOD and CAT, were determined in KO and C57BL/6J mice of different ages in order to evaluate the effect of SELENOF KO on redox homeostasis. Compared with C57BL/6J mice, the content of serum MDA, an end product of lipid peroxidation, significantly increased (*p* < 0.05) in KO mice aged 16 weeks. Serum GSH content significantly decreased (*p* < 0.01) while serum GSSG content did not change in KO mice aged 12 weeks. This resulted in a GSH/GSSG value, an important parameter of oxidative stress [32], that was reduced by 25% (*p* < 0.05) in KO mice aged 12 weeks compared with C57BL/6J mice. In addition, GPX activity decreased significantly in KO mice at 12 weeks old, and T-SOD activity decreased significantly in KO mice at 16 and 21 weeks old. There was no difference in CAT activity between KO and C57BL/6J mice of all ages (Table 1). 

We further detected the oxidative stress parameters in the livers of KO and C57BL/6J mice of different ages. Compared with C57BL/6J mice, MDA content increased significantly (*p* < 0.05) in the livers of KO mice aged 12 weeks. Accordingly, GSH content significantly decreased (*p* < 0.05), while GSSG content significantly increased (*p* < 0.05), leading to a marked reduction in the GSH/GSSG value in KO mice aged 12 weeks. Moreover, hepatic GPX activity in KO mice was much lower (*p* < 0.01) than that in C57BL/6J mice aged 12 weeks. There was no difference in the activities of CAT and SOD between KO and C57BL/6J mice of different ages (Table 2).

**Figure 7:**
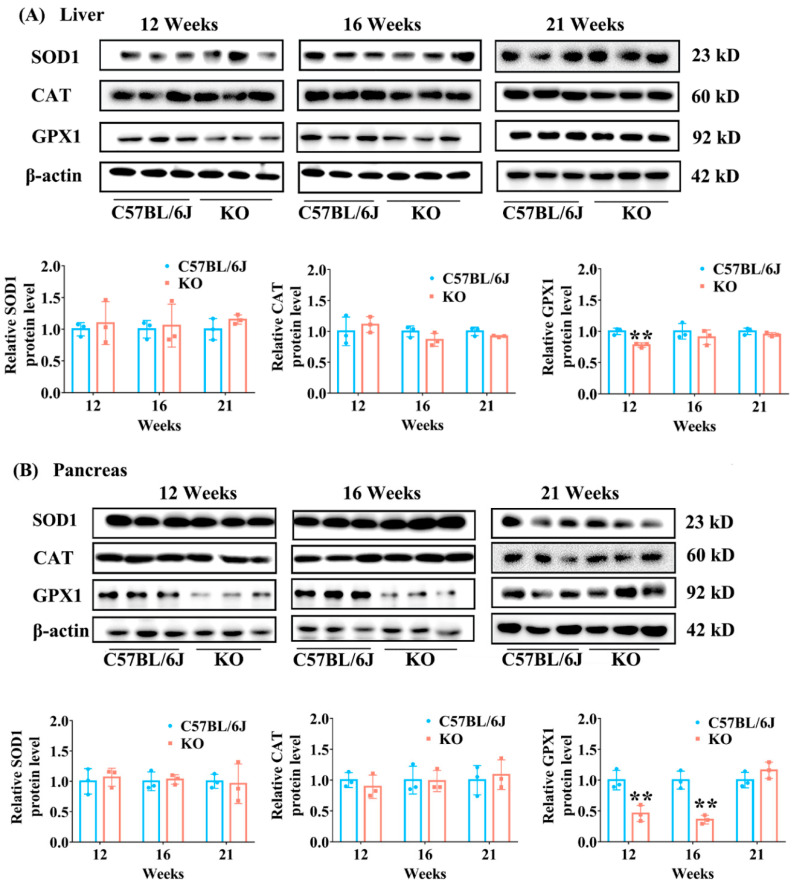
The expression levels of SOD1, CAT and GPX1 in the liver and pancreas of KO and C57BL/6J mice of different ages. (**A**) Representative Western blot bands of SOD1, CAT and GPX1 (upper panel) and their semi-quantification analysis normalized to β-actin (lower panel) in liver tissues. (**B**) Representative Western blot bands of SOD1, CAT and GPX1 (upper panel) and their semi-quantification analysis normalized to β-actin (lower panel) in pancreas tissues. SOD1: Cu/Zn SOD; GPX1: cellular glutathione peroxidase. Data were expressed as mean ± SD (*n* = 3). ** *p* < 0.01, compared with C57BL/6J mice.

The expression of antioxidant enzymes (GPX1, SOD1 and CAT) in the liver and pancreas of KO and C57BL/6J mice was determined to further confirm the effect of SELENOF KO on redox homeostasis. GPX1, the most abundant selenoprotein in mammals, plays an important role in antioxidant defense by reducing H_2_O_2_ to water and lipid hydroperoxides to their corresponding alcohols [14]. SELENOF KO significantly inhibited GPX1 expression (*p* < 0.01) in both the liver and pancreas of mice before 16 weeks old (Figure 7) while the expression of SOD1 and CAT in the liver and pancreas showed no difference between KO and C57BL/6J mice of all ages.

Taken together, the above results indicate that SELENOF KO could disturb redox homeostasis in mice younger than 16 weeks of age leading to oxidative stress.

### 3.7. SELENOF KO Increased the Expression of Hepatic SELENOS in Young Mice

**Figure 8:**
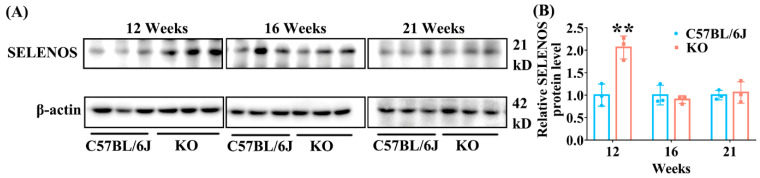
The expression of hepatic SELENOS in KO and C57BL/6J mice of different ages. (**A**) Representative Western blot bands of SELENOS. (**B**) Semi-quantification analysis of SELENOS to β-actin. Data were expressed as mean ± SD (*n* = 3). SELENOS: Selenoprotein S. ** *p* < 0.01, compared with C57BL/6J mice.

Besides GPX1, the expression of the other three selenoproteins, SELENOS, TXNRD1 and SELENOT, in the liver were determined using Western blot analysis. The data showed that hepatic SELENOS level significantly increased in KO mice aged 12 weeks, but did not change in KO mice aged 16 and 21 weeks, in comparison with C57BL/6J mice (Figure 8). The levels of TXNRD1 and SELENOT did not change in the livers of KO mice at all ages (Figure S2).

### 3.8. SELENOF KO Might Not Cause ER Stress in the Liver and Pancreas of Mice with Different Ages

SELENOF has been reported to be involved in the protein quality control process by binding with UGGT and preventing the accumulation of unfolded or misfolded proteins in ER [17,18,19,20]. Excessive accumulation of unfolded or misfolded proteins can cause ER stress, which triggers the death of pancreatic islet β-cells, insulin resistance in skeletal muscle and the liver, and diabetes [33,34]. We analyzed the protein levels of CNX, UGGT and GRP78, three ER-resident chaperone proteins, as markers of ER stress. As shown in Figure S3, SELENOF KO had no significant effect on the expression of these three proteins in both the liver and pancreas of mice at different ages, except a decrease in CNX level (probably degradation of CNX by proteasomes) in the liver of mice aged 21 weeks. Furthermore, HE staining analysis showed no obvious damage in either the liver (Figure S4) or pancreas in the KO mice (Figure S5). These results suggest that SELENOF KO might not have caused ER stress in the liver and pancreas of mice under the conditions of this experiment.

## 4. Discussion

An increasing number of studies have linked SELENOF gene polymorphisms and SELENOF dysregulation to various diseases, including several types of cancers, AIDS, and neurodegeneration [17], but little is known about its association with glucose metabolism and related diseases. We have previously reported on glucose metabolism disorders in SELENOF knockout mice [22,23], however, the underlying mechanism and whether the effect of SELENOF on glucose metabolism is age-dependent remain unknown. In the present work, we investigated the effect of SELENOF KO on glucose metabolism in more detail. The results showed that SELENOF KO caused glucose metabolism disorders only in young mice, especially in 12-week-old mice, characterized by hyperglycemia, serum insulin reduction, impaired glucose tolerance, decreased insulin sensitivity, decreased glucose catabolism, increased gluconeogenesis and impaired AKT signaling pathways. These abnormalities gradually improved with age and disappeared in KO mice at 21 weeks old. Additionally, SELENOF KO mice showed an increased lipid peroxidation, a decreased GSH/GSSG value, and reduced GPX activity and expression before 16 weeks old, suggesting that SELENOF knockout disturbed redox homeostasis. We also found that SELENOF KO caused an increase in hepatic SELENOS expression, a hallmark of type 2 diabetes, in 12-week-old mice. These findings suggest that SELENOF KO might cause the disruption of redox homeostasis and an increase in hepatic SELENOS expression, thus contributing to glucose metabolism disorders in young mice. This interesting facet of SELENOF as a glucose metabolism regulator could broaden its therapeutic potential for glucose metabolism-related diseases such as obesity and diabetes mellitus.

Our results suggested that SELENOF was involved in the regulation of redox homeostasis, especially in young mice. In the present work, SELENOF KO mice younger than 16 weeks of age showed increased lipid peroxidation, a decreased GSH/GSSG value and GPX activity in the serum and liver (Table 1 and Table 2). Furthermore, GPX1 expression in the liver and pancreas of KO mice was decreased significantly compared with C57BL/6J mice (Figure 7). These data suggested oxidative stress in SELENOF KO mice younger than 16 weeks of age. Consistent with our results, Kasaikina et al., have reported mild oxidative stress in the liver of SELENOF KO mice (but they did not specify the age of the mice) [35]. Therefore, SELENOF might play a role in the regulation of redox homeostasis. This role of SELENOF might be attributed to its thiol-disulfide oxidoreductase activity. SELENOF has a thioredoxin-like domain containing a redox active CXU (C is cysteine, U is selenocysteine and X is another residue) motif, resembling the redox motif (CXXC) of thiol-disulfide oxidoreductases [36]. Recently, we obtained multi-milligrams of human SELENOF by chemical protein synthesis. The thiol-disulfide oxidoreductase activity of SELENOF was further supported by its ability to catalyze the reduction and isomerization of disulfide bonds. Moreover, we found that the presence of Sec in the redox motif was the key for this activity [37].

Oxidative stress is one of the factors that causes β-cell damage and/or dysfunction in the pancreas, causing insulin secretion decrease [38,39,40,41]. Correspondingly, our study also found that SELENOF KO mice had significantly lower serum insulin concentration compared with C57BL/6J mice aged 12 weeks (Figure 1D), which was probably due to oxidative damage to the islet β-cells. Insulin is an important hormone that the body needs to maintain blood glucose homeostasis by regulating glucose metabolism, including the glucose catabolism, gluconeogenesis and glycogenesis pathway [42,43]. Once insulin level decreases, this homeostasis will be disrupted. Followed by serum insulin level reduction, we observed that glycolytic enzyme expression (PFK, G6PD) decreased, and gluconeogenesis enzyme expression (FBP) increased (Figure 4) in the liver, thus leading to an increase in fasting blood glucose level in young KO mice (Figure 1C). Another distinct feature that accompanies the reduction of serum insulin level was that the mice were less able to tolerate glucose. Likewise, KO mice exhibited obvious glucose intolerance at 10 weeks old in our study (Figure 2A). Therefore, the disorder of glucose metabolism in young KO mice might be partially attributed to the pancreatic redox homeostasis imbalance caused by SELENOF KO.

Besides β-cell damage and/or dysfunction, oxidative stress is also one of the important triggers of insulin resistance [44,45,46]. During insulin signaling transduction, insulin stimulates autophosphorylation of the insulin receptor thereby phosphorylating and activating insulin receptor substrate, which subsequently activates phosphatidylinositol 3-kinase (PI3K) as well as the downstream phosphoinositide-dependent protein kinase-1 (PDK1) and AKT [47]. AKT activation can further induce glycogen synthesis through increasing phosphorylation of GSK3β and inhibit gluconeogenesis through increasing phosphorylation of FoxO1 [47]. A certain degree of oxidative stress can reduce insulin sensitivity by suppressing the AKT signaling pathway leading to insulin resistance [45,48]. Once the phosphorylation of AKT is inhibited, the phosphorylation levels of GSK3β and FoxO1 also decrease, which causes decreased glycogen synthesis and increased gluconeogenesis [48,49]. Therefore, decreased insulin sensitivity in mice is often accompanied by reduced expression levels of phosphorylated AKT, FoxO1 and GSK3β (normalized to unphosphorylated AKT, FoxO1 and GSK3β, respectively) [49,50]. Consistently, we found that the levels of phosphorylated AKT, FoxO1 and GSK3β (normalized to unphosphorylated AKT, FoxO1 and GSK3β, respectively) were all significantly inhibited in the livers of KO mice aged 12 weeks (Figure 6), and insulin sensitivity was reduced in KO mice aged 16 weeks (Figure 3). Also, increased gluconeogenesis enzyme expression (FBP) and decreased glycogen accumulation was observed in the liver and muscle of KO mice before 16-weeks old (Figure 4 and Figure 5). It should be noted that the relative reduction of phosphorylated AKT and FoxO1 level (normalized to unphosphorylated AKT and FoxO1, respectively) in the livers of KO mice aged 12 weeks was due to the elevation of unphosphorylated AKT and FoxO1 level when normalized to β-actin. To our knowledge, the elevation of unphosphorylated AKT and FoxO1 level is rare in the literature. We speculated that the mRNA expression or degradation of unphosphorylated AKT and FoxO1 might be affected under the condition of SELENOF KO.

We also analyzed the expression of the other three selenoproteins, SELENOS, TXNRD1 and SELENOT in the livers of KO and C57BL/6J mice. SELENOS, another selenoprotein localized in the ER, has been reported to be closely associated with insulin resistance and type 2 diabetes [51,52]. The hepatic SELENOS level increased more significantly in diabetic *P. obesus* than in nondiabetic controls after fasting [51]. Also, increased expression of SELENOS could contribute to insulin resistance in the liver, characterized by reduced glucose uptake, basal and insulin-stimulated glycogen synthesis, and glycogen content [52]. In this work, we found that SELENOS was significantly upregulated in the livers of 12-week-old KO mice but did not change in 16- and 21-week-old KO mice compared with C57BL/6J mice (Figure 8). Being a major protein disulfide reductase in the cell, TXNRD1 is involved in the control of antioxidant defense [53]. Moreover, SELENOT, also an ER-resident selenoprotein, has been reported to be involved in the regulation of glucose metabolism [54,55]. However, we found that the expression levels of TXNRD1 and SELENOT did not change in the livers of KO mice at all ages (Figure S2), suggesting that SELENOF KO had no significant effect on their expression. Therefore, our results suggested that SELENOS expression might be specifically affected by SELENOF KO and the glucose metabolism disorder observed in young SELENOF KO mice might be partly due to the abnormal elevation of SELENOS expression.

SELENOF might be involved in the protein folding quality control process by binding with UGGT and then preventing the accumulation of unfolded or misfolded proteins in ER [17,18,19,20]. Excessive accumulation of unfolded or misfolded proteins can cause ER stress, which triggers the death of pancreatic islet β-cells and insulin resistance in skeletal muscle and the liver in diabetes [33,34]. In the present study, SELENOF KO had no significant effect on the expression of UGGT and GRP78 in either the liver or the pancreas of mice at different ages, except for the decrease in CNX level in the liver of mice aged 21 weeks (Figure S3). A previous study reported that extensive unfolded protein response might lead to protein degradation [19]. Thus, the abnormality of CNX may be due to its degradation by proteasomes. Furthermore, HE staining analysis showed no obvious damage in either the liver (Figure S4) or the pancreas in KO mice (Figure S5). These results suggest that SELENOF KO might not have caused ER stress in the liver and pancreas of mice under the conditions of this experiment.

It should be noted that there are still some limitations in our study. Firstly, we used C57BL/6J mice purchased from a commercial vendor to compare data obtained from SELENOF KO mice. As the C57BL/6J mice and KO mice are from two different sources, there are several factors that may influence the obtained findings (e.g., diets, microbial environment, housing temperature, litter sizes, weights, etc.). Using wild-type littermates as the control group could more accurately reflect the effect of SELENOF on glucose metabolism. Secondly, only male mice were selected for the experiment, and female mice were not studied, so the results are not generalizable. Thirdly, we speculated that SELENOF KO might affect glucose metabolism by affecting redox homeostasis, but the specific molecular target has not been precisely elucidated in our work. Finally, previous studies have shown that glucose metabolism will gradually cause homeostasis imbalance as the body ages [56]. Contrary to this, we found that SELENOF KO caused obvious glucose metabolism disorder at an earlier age and gradually recovered, even showing mild improvement with age in our study. This suggests that the regulation of glucose metabolism by SELENOF might be two-sided with aging. However, we have no more evidence for this hypothesis currently. The above shortcomings are also issues that need to be addressed in our future work so that we can understand the function of SELENOF in more detail and develop its potential application in the treatment of glucose metabolism-related diseases.

## 5. Conclusions

**Figure 9:**
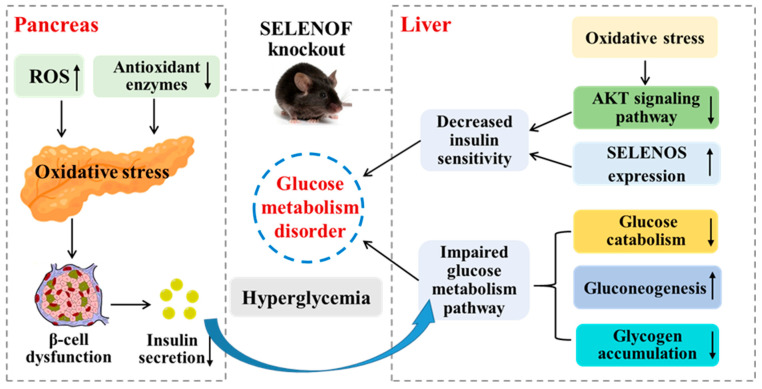
Schematic diagram of glucose metabolism disorders caused by SELENOF KO in young mice and the related mechanism. SELENOF KO could reduce insulin secretion in the pancreas of young mice resulting in impaired glucose metabolism pathways including decreased glucose catabolism and glycogen accumulation as well as increased gluconeogenesis in the liver. SELENOF KO could also reduce insulin sensitivity by inhibiting the AKT signaling pathway and upregulating SELENOS expression in the liver. The redox homeostasis disruption induced by SELENOF KO in the liver and pancreas contributed to glucose metabolism disorders in young mice directly or indirectly.

In this work, we explored the regularity of SELENOF KO’s effect on glucose metabolism in mice and tried to elaborate the possible mechanism. The results indicated that SELENOF KO impaired glucose metabolism in young mice characterized by hyperglycemia, serum insulin reduction, impaired glucose tolerance, decreased insulin sensitivity, decreased glucose catabolism and glycogen accumulation, increased gluconeogenesis and impaired AKT signaling pathway. The mechanism involved may be that SELENOF KO caused an imbalance of redox homeostasis and upregulation of SELENOS expression in the liver and pancreas, both of which led directly or indirectly to glucose metabolism disorder, as illustrated in Figure 9. This study helps us to understand the function of SELENOF in more detail, especially its relationship with glucose metabolism, and suggests that SELENOF may be a potential therapeutic target for diseases caused by glucose metabolism disorder.

## Figures and Tables

**Table 1:** Effects of SELENOF knockout on serum oxidative stress parameters in mice of different ages.

Age (Weeks)	Mice Types	MDA (nmol/mL)	GSH (nmol/mL)	GSSG (nmol/mL)	GSH/ GSSG	GPX (U/mL)	T-SOD (U/mL)	CAT (U/mL)
12	C57BL/6J	11.4 ± 1.0	9.9 ± 1.6	4.3 ± 0.3	2.3 ± 0.3	535.6 ± 90.7	149.3 ± 9.4	16.9 ± 2.2
	KO	12.0 ± 0.7	7.5 ± 1.1 **	4.1 ± 0.5	1.8 ± 0.3 *	350.6 ± 33.8 **	138.4 ± 5.6	15.6 ± 2.5
16	C57BL/6J	10.5 ± 1.0	10.3 ± 2.1	5.2 ± 0.7	2.0 ± 0.3	422.0 ± 85.1	141.8 ± 5.4	14.2 ± 2.1
	KO	12.9 ± 1.1 *	8.6 ± 1.7	5.0 ± 1.0	1.7 ± 0.3	382.5 ± 35.6	127.6 ± 7.1 *	14.5 ± 2.3
21	C57BL/6J	11.5 ± 1.3	10.0 ± 1.8	5.5 ± 0.5	1.8 ± 0.4	438.5 ± 69.0	148.1 ± 6.2	13.4 ± 2.1
	KO	11.8 ± 1.4	8.3 ± 1.8	5.1 ± 0.5	1.6 ± 0.3	395.6 ± 69.7	125.6 ± 8.4 *	13.1 ± 1.9

The data were expressed as mean ± SD (*n* = 8). MDA: malondialdehyde; GSH: glutathione; GSSG: glutathione disulfide; GPX: glutathione peroxidase; T-SOD: total superoxide dismutase; CAT: catalase. * *p* < 0.05, ** *p* < 0.01, compared with WT mice.

**Table 2:** Effects of SELENOF knockout on hepatic oxidative stress parameters in mice of different ages.

Age (Weeks)	Mice Types	MDA (nmol/ mg Protein)	GSH (nmol/mg Protein)	GSSG (nmol/mg Protein)	GSH/ GSSG	GPX (U/mg Protein)	CAT (U/mg Protein)	T-SOD (U/mg Protein)
12	C57BL/6J	1.8 ± 0.3	2.27 ± 0.25	0.26 ± 0.04	8.8 ± 1.5	396.8 ± 36.2	165.8 ± 39.1	311.3 ± 32.3
	KO	2.2 ± 0.3 *	1.93 ± 0.29 *	0.35 ± 0.05 *	5.6 ± 0.7 **	336.4 ± 40.8 **	175.6 ± 36.9	284.5 ± 23.9
16	C57BL/6J	1.6 ± 0.3	2.19 ± 0.45	0.38 ± 0.06	5.8 ± 0.9	410.3 ± 43.8	169.3 ± 38.8	284.1 ± 22.8
	KO	1.9 ± 0.4	2.02 ± 0.37	0.39 ± 0.07	5.3 ± 1.1	374.8 ± 41.1	182.0 ± 37.8	309.7 ± 31.2
21	C57BL/6J	1.8 ± 0.3	2.60 ± 0.38	0.50 ± 0.06	5.2 ± 0.9	430.3 ± 90.3	186.9 ± 43.6	254.8 ± 34.6
	KO	2.0 ± 0.4	2.30 ± 0.21	0.45 ± 0.05	5.1 ± 0.6	382.9 ± 41.4	183.6 ± 41.6	278.5 ±30.8

The data were expressed as mean ± SD (*n* = 8). * *p* < 0.05, ** *p* < 0.01, compared with C57BL/6J mice.

## Data Availability

All of the data is contained within the article and the supplementary materials.

## Supplementary Materials

The following are available online at https://www.mdpi.com/article/10.3390/antiox11112105/s1, Table S1: Antibodies used in Western blot analysis. Scheme S1: The schematic design of animal experiments. Figure S1: Identification of selenoprotein F (SELENOF) knockout (KO) mice of different ages. Figure S2: The expression levels of selenoprotein T (SELENOT) and thioredoxin reductase 1 (TXNRD1) in the liver of C57BL/6J mice and SELENOF KO mice of different ages. Figure S3: The expression levels of key proteins related to protein folding quality control in liver and pancreas tissues of SELENOF KO and C57BL/6J mice of different ages. Figure S4: Representative images of hematoxylin and eosin (HE) staining of the liver from C57BL/6J mice or SELENOF KO mice of different ages. Figure S5: Representative images of HE staining of the pancreas from C57BL/6J mice or SELENOF KO mice of different ages.
