# Supplementary material for: Selenoprotein F Knockout Caused Glucose Metabolism Disorder in Young Mice by Disrupting Redox Homeostasis
Source: Antioxidants (Basel). 2022 Oct 25;11(11):2105. doi: 10.3390/antiox11112105 (PMC9686732; doi:10.3390/antiox11112105)
Supplement: Supplementary file 1 [file antioxidants-11-02105-s001.zip › antioxidants-1972615-supplementary.pdf]

Supplemental information

Selenoprotein F knockout caused glucose metabolism disorder in young mice by Disrupting Redox Homeostasis

Min Li[1], Yun Zhang[2], Jun Zhou[1,3], Hongmei Liu[1,3,*]

[1] Hubei Key Laboratory of Bioinorganic Chemistry and Materia Medica, School of Chemistry and Chemical Engineering, Huazhong University of Science and Technology, Wuhan, 430074, China.

[2] College of Chemistry and Molecular Sciences, Wuhan University, Wuhan, 430072, China.

[3] Shenzhen Huazhong University of Science and Technology Research Institute, Shenzhen, 518057, China.

*Corresponding author. E-mail address: hongmeiliuhust@hust.edu.cn; Tel: +86 27 87543032; Fax: +86 27 87543632.

Table S1 Antibodies used in Western blot analysis

Antibody	Company	Cat number
SOD1	Wanleibio	WL01846
GPX1	Wanleibio	WL02497a
AKT	Wanleibio	WL0003b
p-AKT	Wanleibio	WLP001a
FoxO1	Wanleibio	WL02491
p-FoxO1	Wanleibio	WL03634
GSK3β	Wanleibio	WL01456
p-GSK3β	Wanleibio	WL03518
CNX	Wanleibio	WL03062
GRP78	Wanleibio	WL03157
UGGT	Abcam	ab241357
SELENOT	Abcam	ab176192
SELENOS	Abcam	ab50354
SELENOF	Abcam	ab124820
TXNRD1	Boster	BM5241
G6PD	Beyotime	AF6945
FBP	Beyotime	AF6870
β-actin	Beyotime	AF5001
GAPDH	Beyotime	AF2819
β-Tubulin	Beyotime	AF1216
CAT	GeneTex	GTX110704
PFK	Santa Cruz	sc-393713

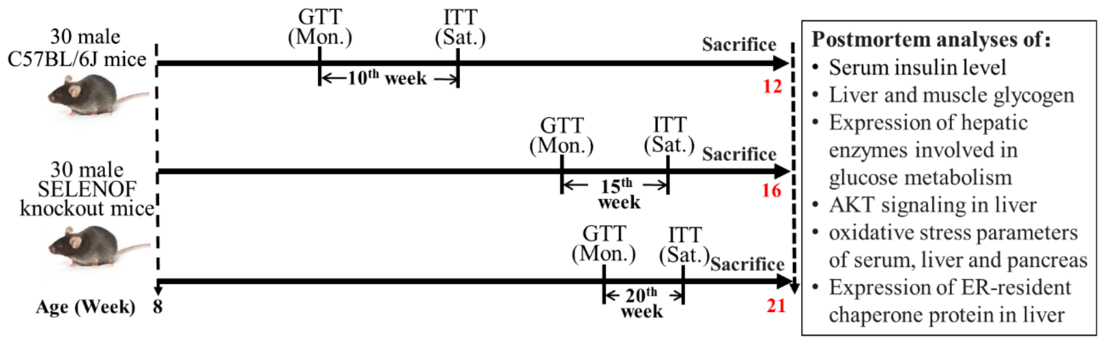

Scheme S1 The schematic design of animal experiments.

(A)

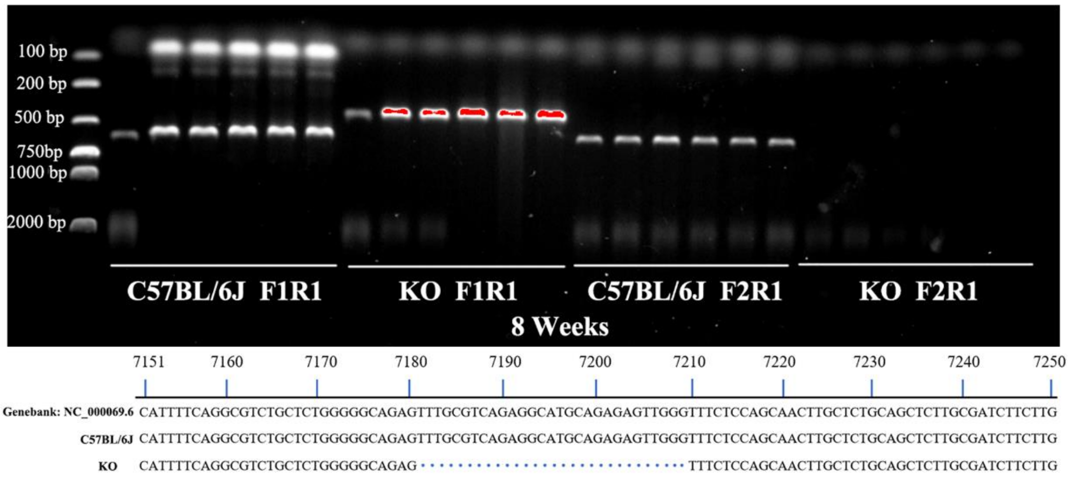

(B)

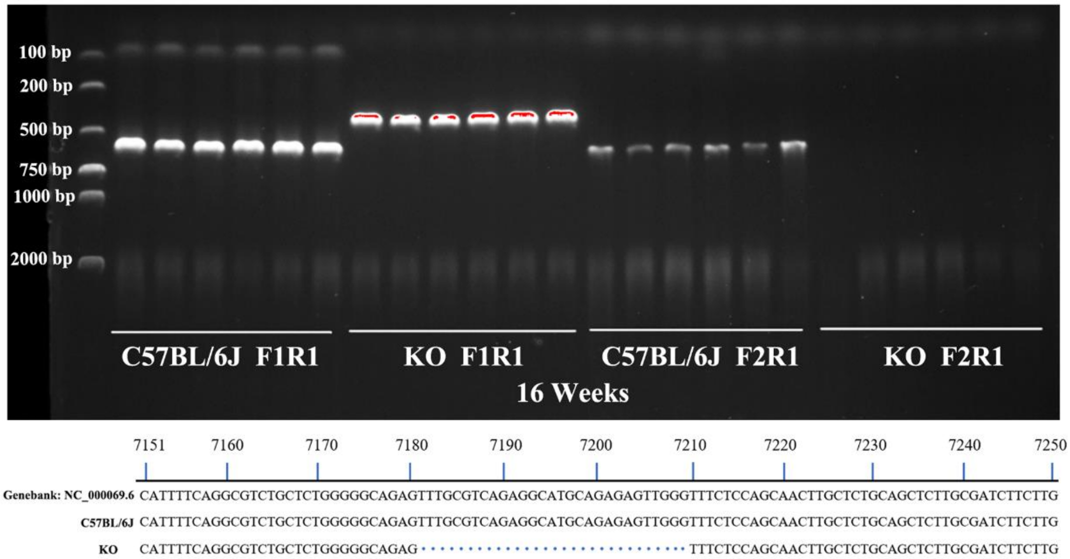

(C)

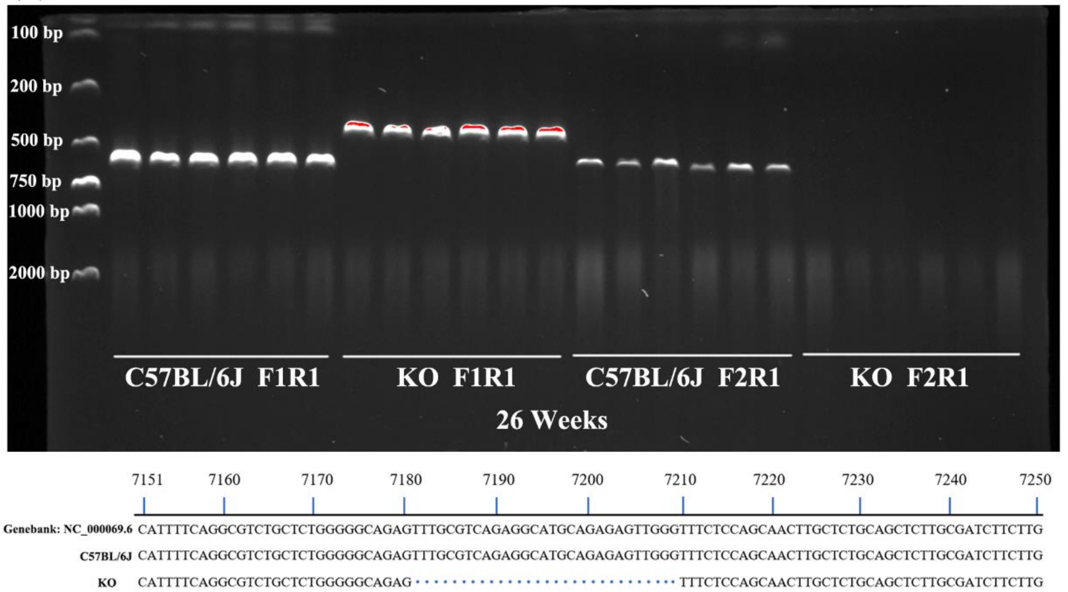

(to be continued)

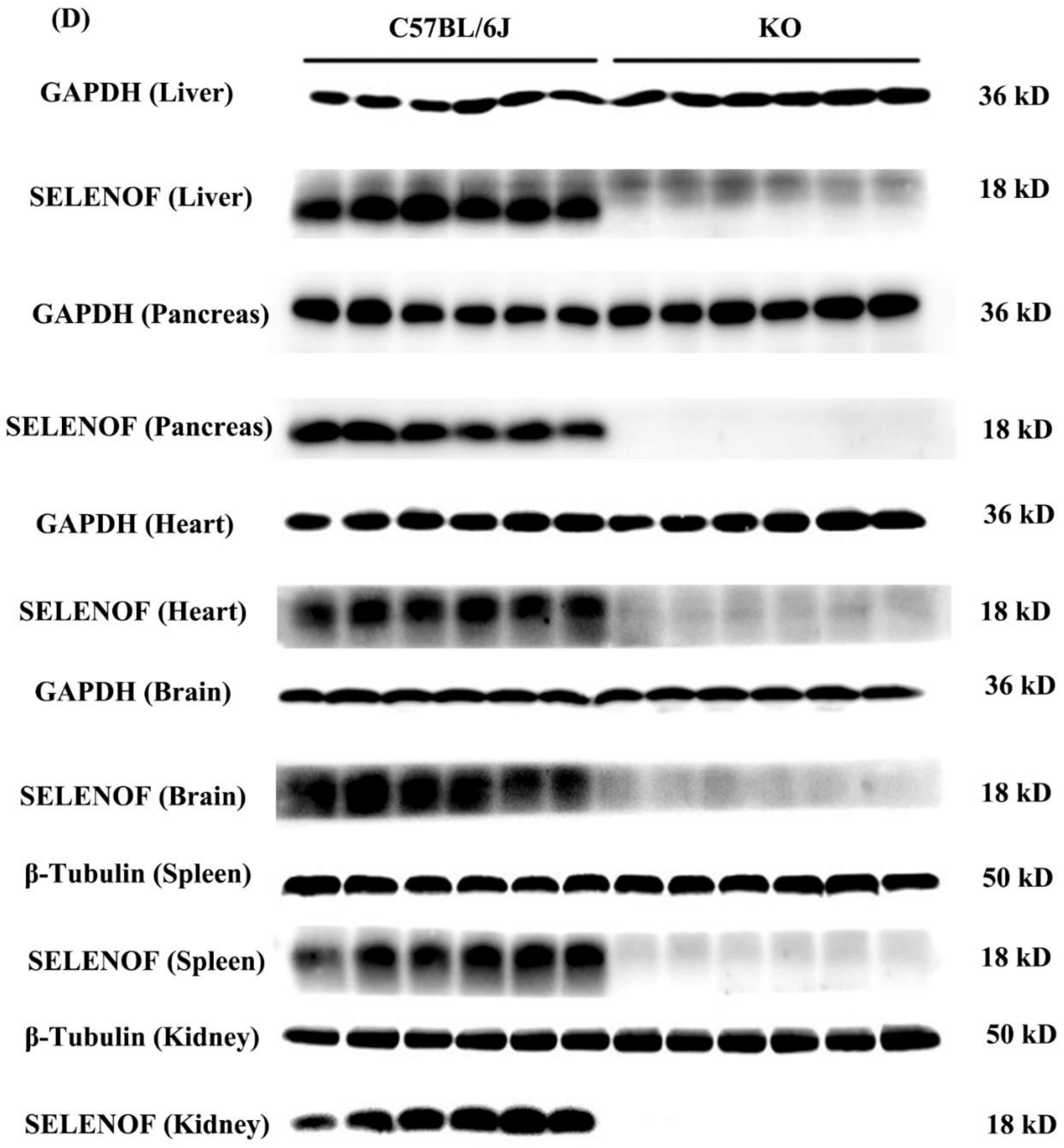

Figure S1. Identification of selenoprotein F (SELENOF) knockout (KO) mice of different ages. DNA were extracted from tail cuts of mice aged 8,16 and 26 weeks using alkali lysis method, and amplified with polymerase chain reaction (PCR). The Forward Primer 1 (F1, 5'-ACAGAGCTAGTTCAGGGCCA-3') and the Reverse Primer 1 (R1, 5'-CCAGGAAGCCTAGGGCAATG-3') were designed across the target sequence of the knockout site so that it can be used as positive control. Sharing the same Reverse Primer with the Forward Primer 1, Forward Primer 2 (F2, 5'-GGCAGAGTTTGCGTCAGA-3') is designed right on the target so that it can be used to identify the KO mice. After amplifiction, the product was detected by agarose gel electrophoresis and DNA suquencing, respectively. SELENOF protein level in some tissues (liver, pancreas, heart, brain, spleen and kidney) from C57BL/6J mice and SELENOF KO mice aged 42 weeks were detected by Western blot. (A-C) Agarose gel electrophoresis and nucleotide sequencing of the PCR products from C57BL/6J mice and SELENOF KO mice aged 8 (A), 16 (B) and 26 (C) weeks, respectively. (D) Representative Western blot bands of SELENOF in some tissues from C57BL/6J mice and SELENOF KO mice aged 42 weeks.

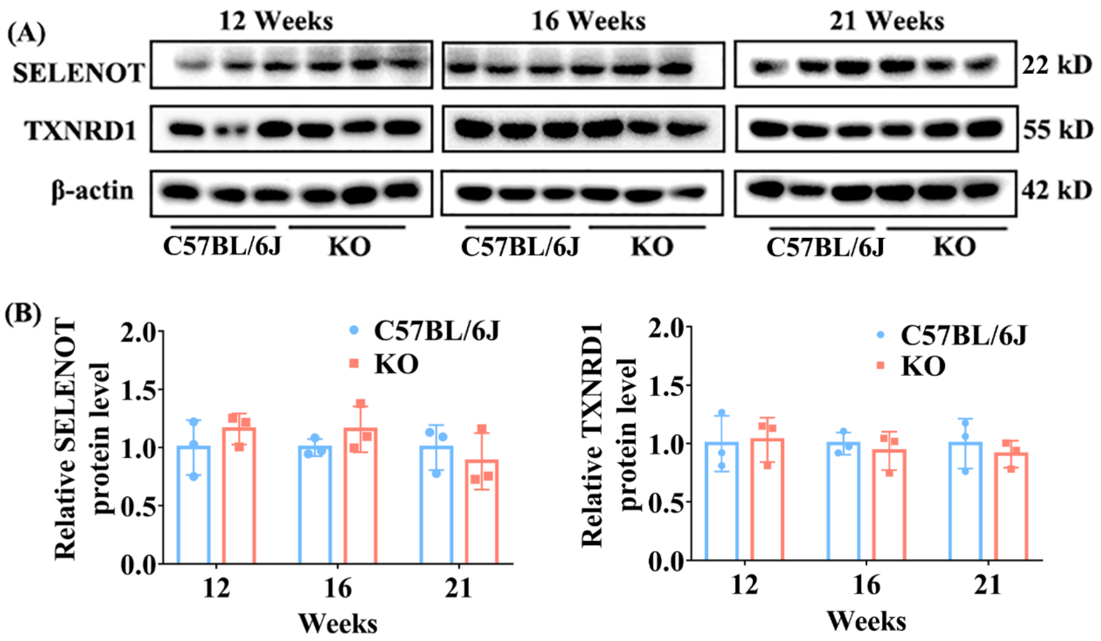

Figure S2. The expression levels of selenoprotein T (SELENOT) and thioredoxin reductase 1 (TXNRD1) in the liver of C57BL/6J mice and SELENOF KO mice of different ages. (A) Representative Western blot bands of SELENOT and TXNRD1. (B) Semi-quantification analysis of SELENOT and TXNRD1 normalized to β-actin (lower panel). Data were expressed as mean ± SD (*n*=3).

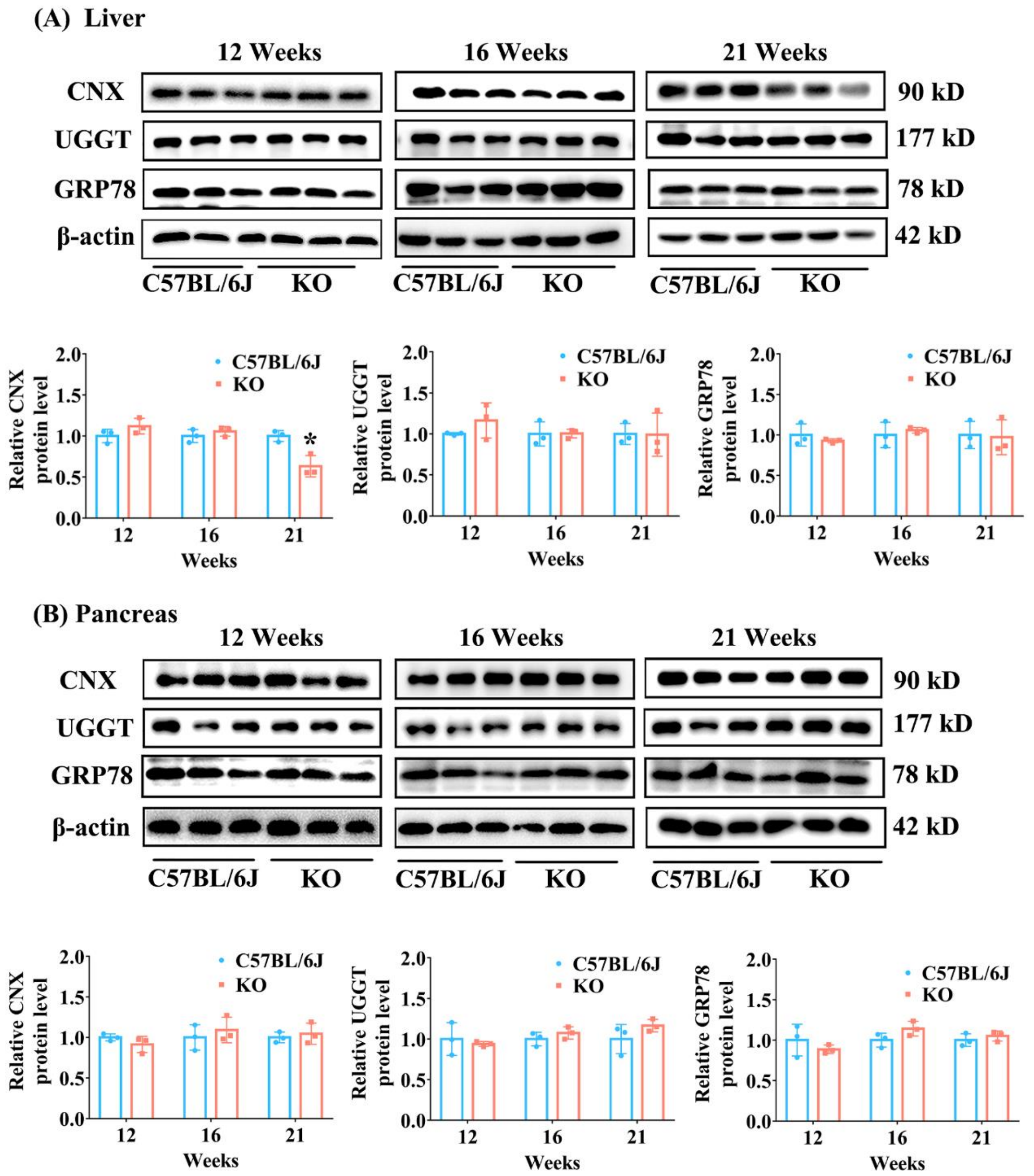

Figure S3. The expression levels of key proteins related to protein folding quality control in liver and pancreas tissues of SELENOF KO and C57BL/6J mice of different ages. (A) Representative Western blot bands of CNX, UGGT and GRP78 (upper panel) and their semi-quantification analysis normalized to β-actin (lower panel) in liver tissues. (B) Representative Western blot bands of CNX, UGGT and GRP78 (upper panel) and their semi-quantification analysis normalized to β-actin (lower panel) in pancreas tissues. CNX: calnexin; UGGT: UDP-glucose: glycoprotein glucosyltransferase; GRP78: glucose regulated protein 78 kD. Data were expressed as mean ± SD (n=3). *$p < 0.05$, compared with C57BL/6J mice.

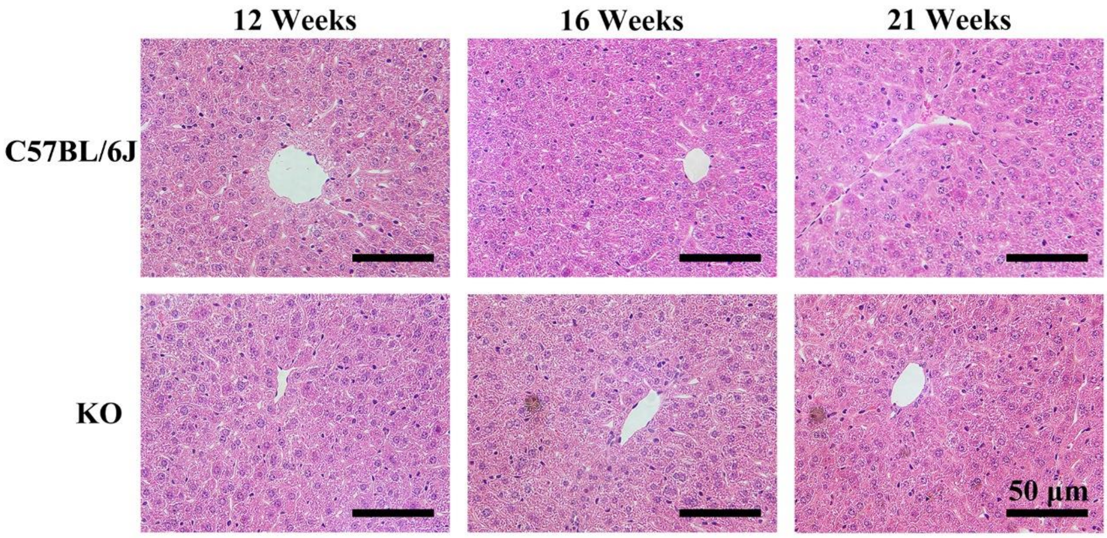

Figure S4. Representative images of hematoxylin and eosin (HE) staining of the liver from C57BL/6J mice or SELENOF KO mice of different ages. Scale bar = 50 μm.

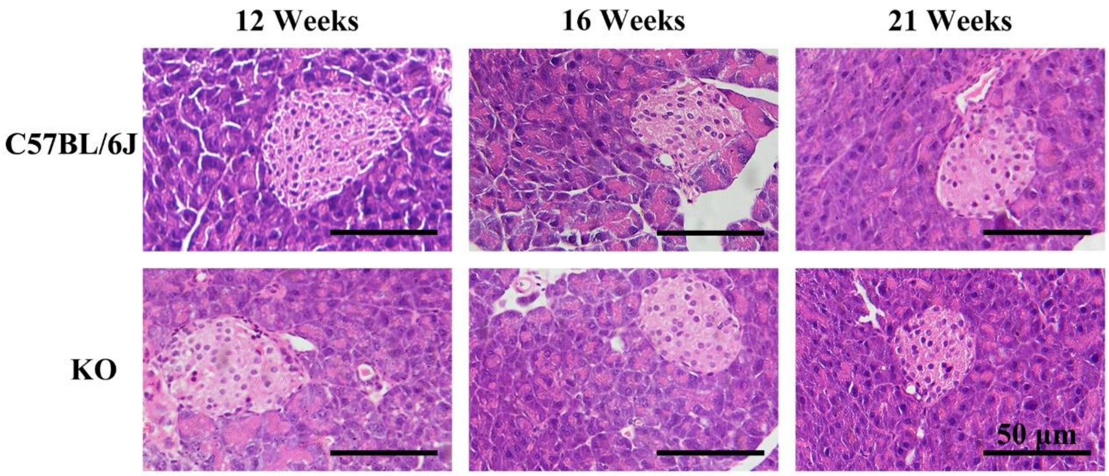

Figure S5. Representative images of HE staining of the pancreas from C57BL/6J mice or SELENOF KO mice of different ages. Scale bar = 50 μm.
